# ROCK activity and the Gβγ complex mediate chemotactic migration of mouse bone marrow-derived stromal cells

**DOI:** 10.1186/s13287-015-0125-y

**Published:** 2015-07-24

**Authors:** Caroline M. Ryan, James A. L. Brown, Emer Bourke, Áine M. Prendergast, Claire Kavanagh, Zhonglin Liu, Peter Owens, Georgina Shaw, Walter Kolch, Timothy O’Brien, Frank P. Barry

**Affiliations:** Regenerative Medicine Institute (REMEDI), Biosciences, National University of Ireland Galway, University Road, Galway, Ireland; Systems Biology Ireland, UCD Conway Institute, University College Dublin, Belfield, Dublin 4, Ireland; Present address: Discipline of Surgery, School of Medicine, Lambe Institute, Translational Research Facility, National University of Ireland Galway, University Road, Galway, Ireland; Discipline of Pathology, School of Medicine, National University of Ireland Galway, University Road, Galway, Ireland; Present address: Hematopoietic Stem Cells and Stress’ group, Division of Stem Cells and Cancer, Deutsches Krebsforschungszentrum (DKFZ), Im Neuenheimer feld 280, 69120 Heidelberg, Germany; Department of Radiology, University of Arizona, Tucson, AZ 85724-5067 USA; Centre for Micro and Imaging, National University of Ireland Galway, University Road, Galway, Ireland

## Abstract

**Introduction:**

Bone marrow-derived stromal cells (BMSCs), also known as mesenchymal stem cells, are the focus of intensive efforts worldwide to elucidate their function and biology. Despite the importance of BMSC migration for their potential therapeutic uses, the mechanisms and signalling governing stem cell migration are still not fully elucidated.

**Methods:**

We investigated and detailed the effects of MCP-1 activation on BMSCs by using inhibitors of G protein-coupled receptor alpha beta (GPCR αβ), ROCK (Rho-associated, coiled-coil containing protein kinase), and PI3 kinase (PI3K). The effects of MCP-1 stimulation on intracellular signalling cascades were characterised by using immunoblotting and immunofluorescence. The effectors of MCP-1-mediated migration were investigated by using migration assays (both two-dimensional and three-dimensional) in combination with inhibitors.

**Results:**

We established the kinetics of the MCP-1-activated signalling cascade and show that this cascade correlates with cell surface re-localisation of chemokine (C motif) receptor 2 (CCR2) (the MCP-1 receptor) to the cell periphery following MCP-1 stimulation. We show that MCP-1-initiated signalling is dependent on the activation of βγ subunits from the GPCR αβγ complex. In addition, we characterise a novel role for PI3Kγ signalling for the activation of both PAK and ERK following MCP-1 stimulation. We present evidence that the Gβγ complex is responsible for PI3K/Akt, PAK, and ERK signalling induced by MCP-1 in BMSCs. Importantly, we found that, in BMSCs, inhibition of ROCK significantly inhibits MCP-1-induced chemotactic migration, in contrast to previous reports in other systems.

**Conclusions:**

Our results indicate differential chemotactic signalling in mouse BMSCs, which has important implications for the translation of in vivo mouse model findings into human trials. We identified novel components and interactions activated by MCP-1-mediated signalling, which are important for stem cell migration. This work has identified additional potential therapeutic targets that could be manipulated to improve BMSC delivery and homing.

**Electronic supplementary material:**

The online version of this article (doi:10.1186/s13287-015-0125-y) contains supplementary material, which is available to authorized users.

## Introduction

Bone marrow-derived stromal cells (BMSCs), also known as mesenchymal stem cells (MSCs), have generated much interest in recent years. Critical properties that contribute to the tissue regenerative capacity of BMSCs include their ability to differentiate into selected cell types, their ability to secrete paracrine factors, and their migratory capacity, which is central to their ability to contribute to a repair response [1–3]. However, the cellular mechanics and chemotactic signalling events that guide BMSCs to their appropriate microenvironments have not been fully elucidated. Fully understanding these mechanisms will advance the therapeutic utility of BMSCs, improving methods for systemic delivery by enhancing efficiency of homing to target tissues. Although it has been previously reported that human BMSCs migrate in response to the chemokine, monocyte chemoattractant protein 1 (MCP-1) [4, 5], the precise mechanisms remain to be completely elucidated. Furthermore, it was shown that MCP-1 migration is mediated by chemokine (C motif) receptor 2 (CCR2) and the adapter molecule Pericentrin-1 (Nup85, FROUNT) [6, 7]. However, the kinetics of CCR2 activation and the downstream pathways involved remain unclear.

Chemotaxis is initiated when chemokines bind to transmembrane receptors, leading to the release of the Gα and Gβγ subunits from the Gαβγ complex of G protein-coupled receptors (GPCRs) [8, 9]. The selective activation of distinct pathways suggests that the GPCRs bind G proteins and additional effectors. Previously, it was demonstrated that Pericentrin-1 binds activated CCR2, linking it to the PI3 kinase (PI3K)-Rac-lamellipodium cascade [7]. However, it is unresolved whether these signalling events are likewise involved in the chemotactic response of BMSCs. Additionally, RhoGTPase family members and the Rho kinase ROCK (Rho-associated, coiled-coil containing protein kinase), which are important mediators of polarisation and migration in many cells types, remain to be confirmed in BMSCs [10–15]. Here, we evaluate the role of ROCK, GPCR, and PI3K signalling events which mediate the chemotactic migration of BMSCs. Using a combination of migration assays, high-resolution imaging, and pharmacological inhibitors, we have explored the critical mediators and order of signalling in MCP-1-mediated BMSC migration.

## Methods

### Cell culture

BMSCs were isolated from 8- to 10-week-old BalbC mice. Marrow was flushed from femurs and tibiae and plated directly onto plastic. BalbC BMSCs were maintained in modified Eagle’s medium-α (MEM-α) GlutaMAX™ (Life Technologies, Carlsbad, CA, USA) supplemented with 10 % fetal bovine serum (HyClone, part of GE Healthcare, Little Chalfont, UK), 10 % equine serum (HyClone), 1 % L-glutamine, and 1 % penicillin-streptomycin. Cells were seeded at a density of 5.7×10^3^ cells/cm^2^, and all experiments were performed by using BMSCs at passages 4–8. Human MSCs were aspirated under sterile conditions from the iliac crests of healthy human volunteers. The obtained marrow was filtered with a 70-μm cell strainer (Falcon, part of Thermo Fisher Scientific, Waltham, MA, USA) before centrifuging at 400×g for 10 min. Cell pellets were resuspended in media consisting of MEM-α (Gibco, part of Invitrogen, Carlsbad, CA, USA), supplemented with 10 % fetal bovine serum (Gibco) and 1 % antibiotics (streptomycin and penicillin) (Gibco), and cultured in 175-cm^2^ flasks at 37 °C in a humidified atmosphere containing 5 % CO_2_. At day 4, the cultures were washed with phosphate-buffered saline to remove the non-adherent cells and further expanded until more than 80 % confluence, when they were harvested and expanded in 175-cm^2^ flasks. After subculture, these cells were designated as passage 1.

BMSCs were characterised by their ability to differentiate into chondrocytes, adipocytes, and osteoblasts and were confirmed to be CD105-, CD73-, Sca1-, CD29-, CD44-positive and CD34-, CD45-, and CD31-negative, as previously described [16]. BMSCs were cultured in serum-free conditions for 2 h prior to pre-treatment with either the CCR2 antagonist: 10 μM RS102895 (Sigma-Aldrich, St. Louis, MO, USA), 10 μM Anti-BetaGamma (MPS-Phosducin-like protein C terminus) (AnaSpec, Fremont, CA, USA) or the PI3K inhibitor: 5 μM LY294002 (Merck, Kenilworth, NJ, USA). For two-dimensional (2D) migration assays, BMSCs were cultured in serum-free conditions overnight prior to assays. BMSCs were pre-treated with ROCK inhibitor (10 μM Y27632; Sigma-Aldrich) in combination with serum-free conditions for 24 h.

All work (both human and animal related) was approved by the Animal Ethics Committee, National University of Ireland, Galway, and conducted under license from the Department of Health, Ireland. Human MSCs were obtained and purified in accordance with the described protocol (above) from fully informed and consenting patients as approved by the Animal Ethics Committee, National University of Ireland, Galway. All animal experiments were conducted in compliance with the “Principles of Laboratory Animal Care” formulated by the National Society for Medical Research and the “Guidelines for the Care and Use of Laboratory Animals” prepared by the National Academy of Science, which was published by the National Institutes of Health (ISBN 0-309-05377-3, revised 1996).

### Migration assays

Three-dimensional (3D) migration in response to MCP-1 (R&D Systems, Minneapolis, MN, USA) through a porous membrane (8-μm pore size) was assessed using the xCELLigence Real Time Cell Analyzer (Roche Applied Science, Basel, Switzerland). The xCELLigence system measures electrical impedance across a high-density electrode array coating the bottom of a well. The impedance values are converted into a migration index (MI) and these values directly correspond to the number of adhered cells [17]. When cells are not present or are not adhered to the electrodes, the MI is 0. Optimisation indicating the linear phase of BMSC migration occurred within 3 h (data not shown), and subsequently all BMSC migration was recorded in real time for 3 h. For 3D migration, MSCs were cultured in serum-free conditions for 4 h prior to all assays. Assays with combinations of the indicated inhibitors were performed for an additional 2 h. BALB/c BMSCs (5×10^4^) suspended in serum-free medium were seeded into the upper wells of the Cell Invasion and Migration plate, and recombinant mouse MCP-1 in serum-free medium was added to the lower wells. Lower wells containing either 0 % (negative) or 10 % (positive) serum were included as controls. 2D migration was assessed by using the μslide chemotaxis^2D^ system (ibidi, Martinsried, Germany) in the presence of a stable linear chemoattractant gradient established over 3 h in accordance with the instructions of the manufacturer. Cells from the 2D migration were visualized as indicated below.

### Immunofluorescence

BMSCs were seeded onto glass slides and cultured for 48 or 24 (2D chemoattraction assays) h prior to the indicated treatments. BMSCs were exposed to 20 ng/ml MCP-1 at 37 °C for the indicated times and fixed in 4 % paraformaldehyde. Cells were permeabilised with 0.1 % TritonX100 for 30 min, unless otherwise indicated. Fixed cells were blocked with 5 % BSA (Sigma-Aldrich) and stained with the following antibodies: anti-CCR2 antibody ([E68] ab32144; Abcam, Cambridge, UK), antiPericentrin-1 antibody (Santa Cruz Biotechnology, Dallas, TX, USA), or mouse monoclonal anti αtubulin (T5168; Sigma-Aldrich). Bound antibodies were detected by using the appropriate secondary antibody: Cy3-labeled anti-rabbit antibody (Jackson ImmunoResearch Europe, Newmarket, Suffolk, UK), FITC labeled anti-goat IgG antibody (Santa Cruz Biotechnology), or Dylight 488labeled anti-mouse antibody. F-Actin was detected by using Rhodamine-conjugated phalloidin (Sigma-Aldrich). Isotype controls were mouse and rabbit (Thermo Fisher Scientific). Slides were mounted with slowfade gold antifade reagent with 4′,6-diamidino-2-phenylindole (DAPI) (Invitrogen). Z-stack images were captured by using an Andor Revolution spinning disk confocal microscope (Andor, Belfast, Northern Ireland, including an Olympus inverted IX 81 microscope, Olympus, Tokyo, Japan, 40× or 60× oil immersion objective lenses with an Andor iXon^EM+^ EMCCD camera). Z-stack images were processed by using Andor IQ 2.3 software, and images were presented as maximum intensity projections.

### Immunoblotting

BMSCs were cultured for 48 h, serum-depleted, and treated with the inhibitors as indicated. Whole cell lysates were prepared by using RIPA buffer containing a protease inhibitor cocktail (Roche, Basel, Switzerland) with an additional 100 mM phenylmethylsulfonyl fluoride (PMSF) and 100 mM NA_3_VO_4_ (Sigma-Aldrich). Whole cell lysates were separated by SDS-PAGE, and proteins were transferred to polyvinylidene difluoride (PVDF) (Immobilon, EMD Millipore Corporation, Billerica, MA, USA) membranes in accordance with the instructions of the manufacturer. Membranes were blocked with 5 % skim milk powder and incubated overnight at 4 °C with the indicated primary antibodies: phosphoAkt^Ser473^ (Cell Signaling Technology, Beverly, MA, USA), AKT (Cell Signaling Technology), phosphoERK1/2 (Cell Signaling Technology), ERK (Cell Signaling Technology), phosphoPAK1^Ser199/204^/phosphoPAK2^Ser192/197^ (Cell Signaling Technology), and PAK1/2/3 (Cell Signaling Technology). Membranes were incubated for 1 h at room temperature with a horseradish peroxidase-conjugated anti-mouse or anti-rabbit IgG secondary antibody (Jackson ImmunoResearch Laboratories Inc., West Grove, PA, USA). Bound antibodies were visualized by using SuperSignal West Pico Chemiluminescent substrate Thermo Fisher Scientific (Waltham, MA, USA), and the band intensities were quantified by using AlphaView software (FluorChem systems; ProteinSimple, San Jose, CA, USA).

### Viability and cytotoxicity assay

Effect of serum-free medium (SFM) treatment, alone or in combination with the indicated inhibitors, on BMSC viability and cytotoxicity was assayed by using the ApoTox-Glo™ Triplex Assay (Promega Corporation, Madison, WI, USA) in accordance with the instructions of the manufacturer, under the indicated conditions.

### Statistical analysis

To avoid any bias, counts were recorded from multiple fields from the indicated number of independent cell culture preparations. Data analyses were performed by using Prism 5 software (GraphPad Software, Inc., La Jolla, CA, USA) or Microsoft Excel (Microsoft Corporation, Redmond, WA, USA). Statistical significance was calculated with a one-way analysis of variance by using a Kruskal-Wallis test with a *post hoc* Dunn’s or Tukey test to evaluate the statistical significance between groups, unless otherwise indicated. A *P* value of less than 0.05 (*) was deemed significant, *P* value of less than 0.01 (**) very significant, and *P* value of less than 0.001 (***) highly significant, as stated in the figure legends. Counts of cell numbers represent at least three separate discrete experiments with more than 100 counts made for each separate independent experiment (n and minimum number measurements for each separate experiment are indicated in figure legends).

## Results

### BMSCs migrate in response to the chemokine MCP-1

**Fig. 1:**
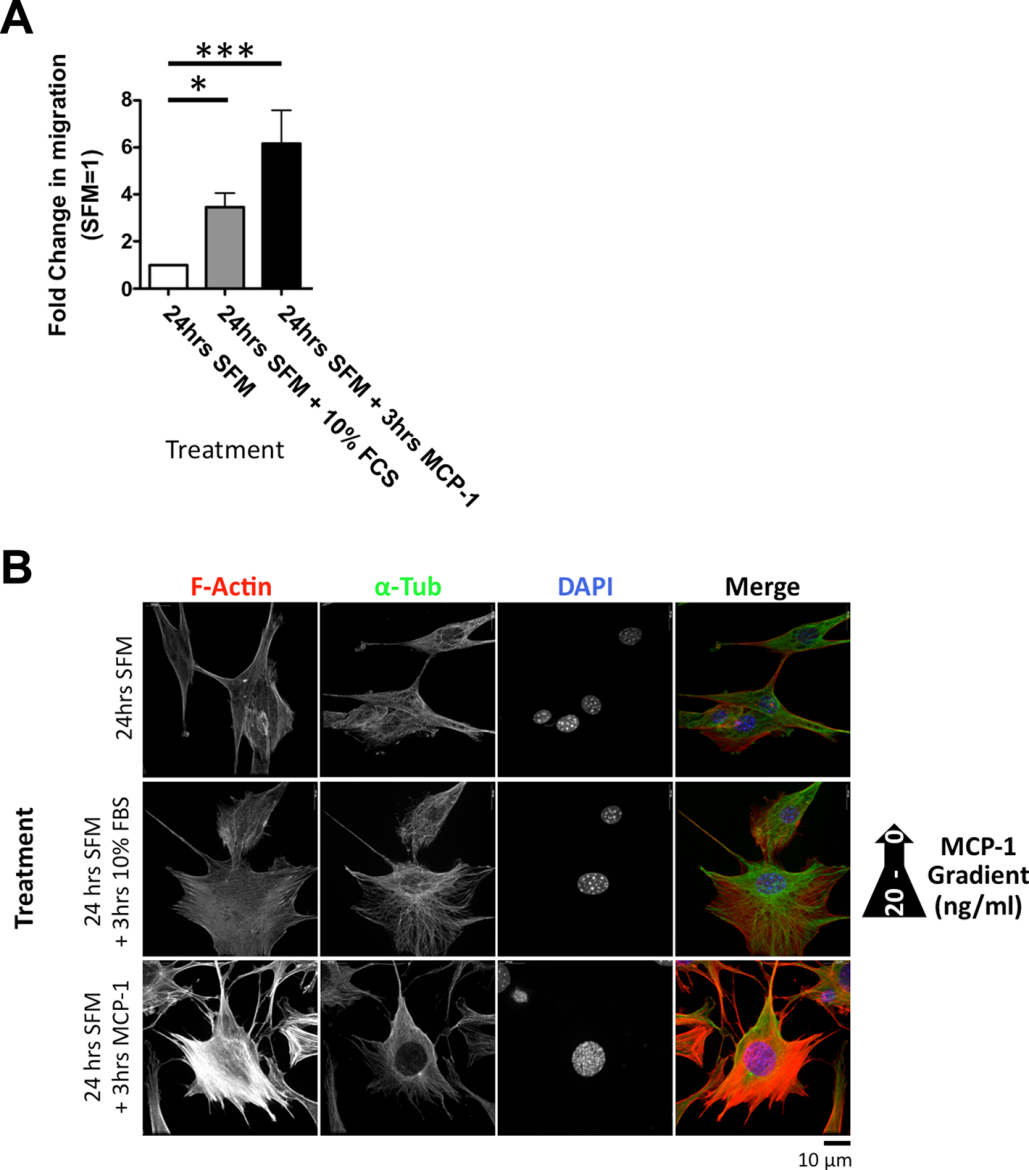
BMSCs migrate and polarise in response to MCP-1. **a** Serum-starved BMSC migration in response to a gradient of 0–20 ng/ml MCP-1 in a two-dimensional system; 24-h pre-treatment with SFM and migration following 3-h exposure to 20 ng/ml MCP-1. SFM was set to 1. Graph represents three independent experiments (>100 cells evaluated for each separate independent experiment). Data are presented as mean ± standard error of the mean. A *P* value of less than 0.05 (*) was deemed significant, and a *P* value of less than 0.001 (***) highly significant. **b** Serum-starved migrating BMSCs polarise in response to MCP-1 treatment. BMSCs immunofluorescently stained with F-actin (red) and α-tubulin (green), following 24-h SFM treatment and 10 % FCS and 20 ng/ml MCP-1 treatment for 3 h. DNA stained with DAPI (blue). Scale bar = 10 μm. *BMSC* bone marrow-derived stromal cell, *DAPI* 4',6-diamidino-2-phenylindole, *FCS* fetal calf serum, *MCP-1* monocyte chemoattractant protein 1, *SFM* serum-free medium

We found that our cultured mouse BMSCs displayed three distinct populations: a tiny immotile fraction (<1 %), polarised BMSCs (~30 %) characterised by an accumulation of actin filaments at the leading edge of the cell, and actively motile BMSCs (~10 %) characterised by defined lamellipodia (concentrated F-actin staining at the cell extension) and an accumulation of α-tubulin at the F-actin-positive cell extension (Additional file 1: Figure S1). The remaining cells observed were characterised as non-polarised, non-migrating. As previously demonstrated [5, 18], MCP-1 induced significant migration (Fig. 1a and Additional file 2: Figure S2a) and polarisation (demonstrated by F-actin re-localisation) in serum-starved mouse BMSCs (Fig. 1b). In addition, a trend (non-significant) of MCP-1 inducing greater migration than the non-specific chemoattractant fetal calf serum (FCS) was observed. Interestingly, investigating the dose-dependent effects of MCP-1 exposure on migration, we found that 20 and 50 ng/ml MCP-1 induced significant migration, compared with the non-specific chemoattractant fetal bovine serum or higher doses of MCP-1 (Additional file 2: Figure S2b). Subsequently, 20 ng/ml MCP-1 was used for all experiments. Supporting previous work demonstrating expression of the MCP-1 receptor gene CCR2 [6, 19], MCP-1 induced significant migration in human BMSCs (Additional file 3: Figure S3a, b). However, in contrast to mouse BMSCs, no difference between FCS- and MCP-1-mediated migration in human BMSCs was observed. Although ROCK inhibitor Y27632 did not inhibit human BMSC MCP-1-mediated migration, it did induce stellate morphology, indicating its effectiveness (Additional file 3: Figure S3a).

### ROCK activity is required for MCP-1-mediated migration in mouse BMSCs

**Fig. 2:**
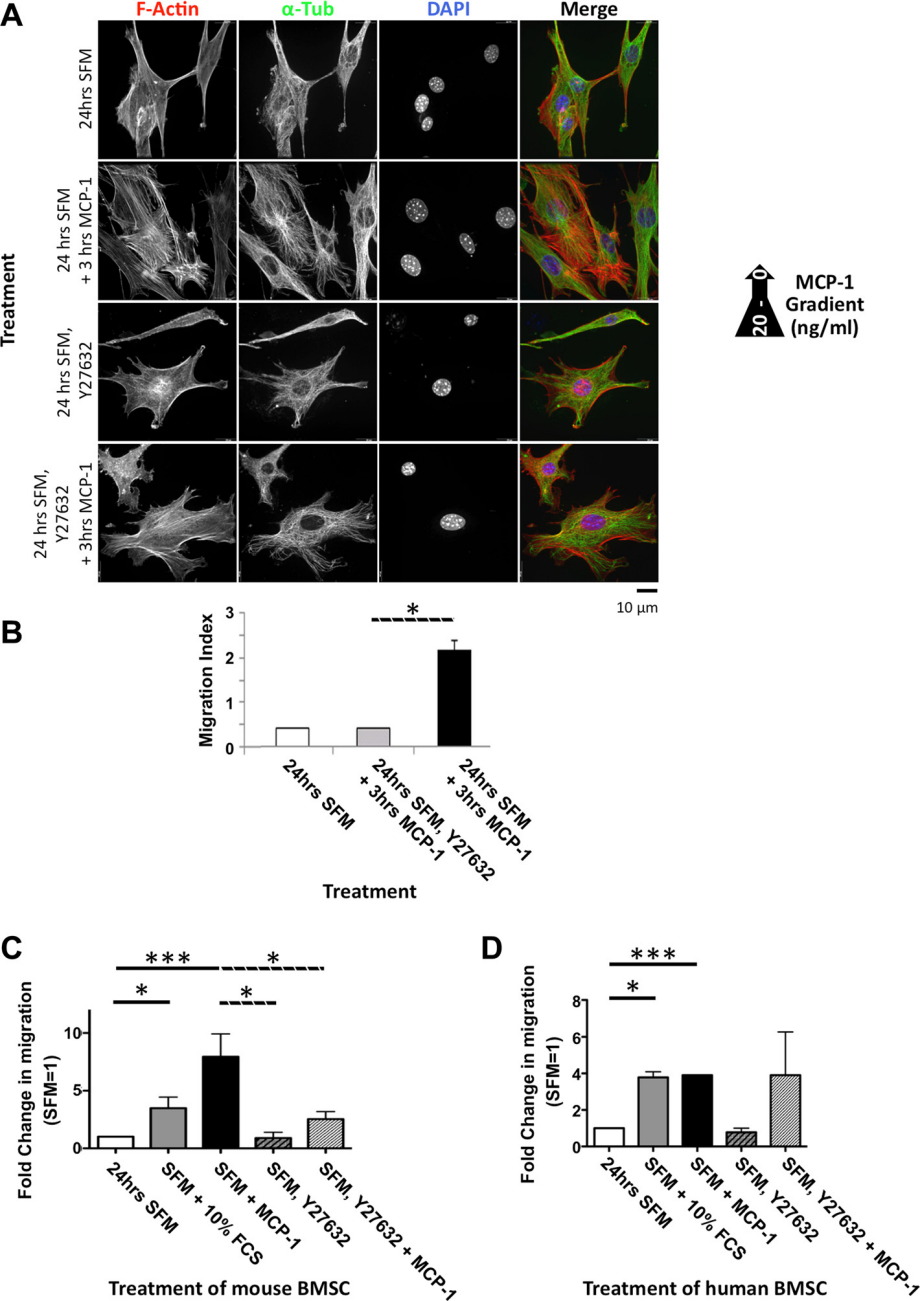
ROCK inhibition significantly decreases BMSC polarisation and migration. **a** ROCK inhibition (10 μM Y27632 pre-treatment for 3 h) significantly reduces serum-starved BMSC polarisation in response to an MCP-1 gradient in a 2D model. Permeabilised serum-starved BMSCs immunofluorescently stained with F-actin (red) and α-tubulin (green) and DNA stained with DAPI (blue), following the indicated treatments. Scale bar = 10 μm. **b**, **c** ROCK inhibition (10 μM Y27632 combined with SFM for 24 h) significantly reduces MCP-1-stimulated BMSC migration. **b** ROCK inhibition (10 μM Y27632 combined with SFM for 24 h) significantly reduces BMSC migration in a three-dimensional assay in response to MCP-1. Graph represents three separate independent experiments. Data are presented as mean ± standard deviation. The migration index (MI) is a relative change in measured electrical impedance. When there are no cells, MI is 0. Increasing MI is a quantitative measure of cells attached to the electrodes, representative of cell numbers migrating though a membrane. **c**, **d** ROCK inhibition (10 μM Y27632 combined with SFM for 24 h) significantly reduces BMSC 2D migration in mouse (**c**) but not human (**d**) cells in response to 3-h MCP-1 exposure. SFM set = 1. Graphs represent three independent experiments (>100 cells evaluated for each separate independent experiment). Data are presented as mean ± standard error of the mean. A *P* value of less than 0.05 (*) was deemed significant, and a *P* value of less than 0.001 (***) highly significant. *2D* two-dimensional, *BMSC* bone marrow-derived stromal cell, *DAPI* 4',6-diamidino-2-phenylindole, *MCP-1* monocyte chemoattractant protein 1, *ROCK* Rho-associated, coiled-coil containing protein kinase, *SFM* serum-free medium

So far, the effect of ROCK inhibition on chemotactic-induced migration has not been examined in mouse BMSCs. Consistent with previous reports, Y27632 pre-treatment resulted in mouse BMSCs with a stellate morphology (Additional file 4: Figure S4a), characteristic of inhibited RhoA/ROCK signalling [20–22]. Furthermore, MCP-1 treatment of Y27632 pre-treated cells did not affect this stellate morphology (Additional file 4: Figure S4a). To investigate the effects on chemotactic migration, BMSCs were cultured in the presence or absence of the ROCK inhibitor, Y27632, for 24 h prior to exposure to an MCP-1 gradient in a 2D migration assay (Fig. 2a and Additional file 4: Figure S4b). We found that Y27632 pre-treatment of mouse BMSC resulted in a significant inhibition in MCP-1-induced migration in both a 3D (Fig. 2b) and 2D migration assay (Fig. 2c). Significantly, however, pre-treatment of human BMSCs with Y27632 did not inhibit MCP-1-mediated migration in the 2D assay (Fig. 2d). Interestingly, human BMSCs treated with Y27632 also displayed a stellate morphology (Additional file 3: Figure S3a, b).

### MCP-1-dependent signalling in mouse BMSCs

**Fig. 3:**
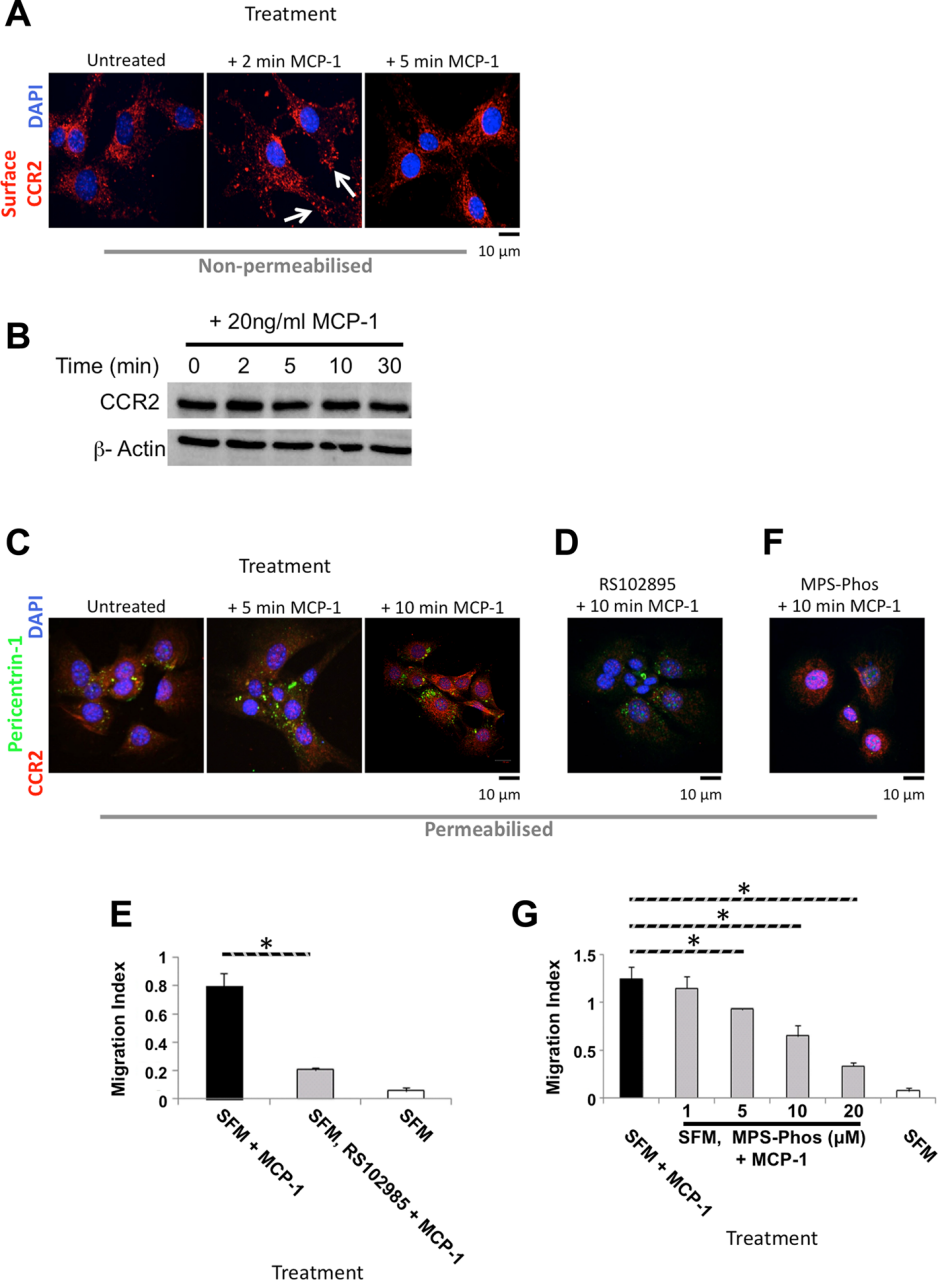
CCR2 and GPCR βγ activity is required for MCP-1-mediated migration. **a** Surface CCR2 re-localises after MCP-1 treatment. Immunofluorescent staining of non-permeabilised serum-starved BMSCs, following the indicated treatments at the indicated times. CCR2 (red). α-tubulin (green). DNA stained with DAPI (blue). Scale bar = 10 μm. **b** Immunoblotting of BMSC whole cell lysates for CCR2 and β-actin following MCP-1 treatment; 100 μg of total cell extract/lane. Representative blot of three separate independent experiments is shown. **c** Kinetics of Pericentrin-1 re-localisation after MCP-1 treatment. Immunofluorescent staining of permeabilised serum-starved BMSCs. CCR2 (red) and Pericentrin-1 (green). DNA stained with DAPI (blue). Scale bar = 10 μm. **d** Serum-starved BMSCs pre-treated with 10 μM RS102895 (βγ GPCR subunit inhibitor) and stained after exposure to MCP-1. CCR2 (red). α-tubulin (green). DNA stained with DAPI (blue). Scale bar = 10 μm. **e** 24-h treatment with 10 μM RS102895 combined with serum starvation inhibits MCP-1-mediated BMSC migration in 3D. Graph represents three separate independent experiments. Data are presented as mean ± SD. **f** MCP-1-mediated Pericentrin-1 re-localisation is inhibited by 24-h pre-treatment with 10 μM MPS-Phos (GPCR βγ inhibitor) and SFM. Permeabilised BMSCs stained with CCR2 (red) and α-tubulin (green). DNA stained with DAPI (blue). Scale bar = 10 μm. **g** Combination of serum-starved BMSCs with a GPCR βγ inhibitor (10 μM MPS-Phos) for 24 h significantly reduced MCP-1-mediated migration in 3D. Graph represents three separate independent experiments. Data are presented as mean ± SD. A *P* value of less than 0.05 (*) was deemed significant. *3D* three dimensions, *BMSC* bone marrow-derived stromal cell, *CCR2* chemokine (C motif) receptor 2, *DAPI* 4′,6-diamidino-2-phenylindole, *GPCR* G protein-coupled receptor, *MCP-1* monocyte chemoattractant protein 1, *MPS-Phos* MPS-phosducin-like protein C terminus, *SD* standard deviation, *SFM* serum-free medium

As chemotactic migration in mouse BMSCs was inhibited by Y27632 treatment, in contrast to previous migration studies [12, 14, 20, 23–25], we characterised the MCP-1-dependent migration signalling pathway in mouse BMSCs. Investigating localisation of the extra-cellular MCP-1 receptor CCR2 (a member of the G protein-coupled receptor superfamily) on the surface of non-permeabilised untreated mouse BMSCs, we found that CCR2 displayed diffuse generalised cell surface staining (Fig. 3a). To support previous studies, we carried out a more detailed analysis, characterising the kinetics of this re-localisation. After 2 min of MCP-1 treatment, surface CCR2 re-distributed to the cell periphery and displayed a distinct punctate staining pattern (Fig. 3a, white arrows). Confirming a re-distribution of CCR2, we demonstrated no change in CCR2 levels following MCP-1 treatment at the indicted time points (Fig. 3b).

To further examine MCP-1-dependent signalling, the CCR2 adaptor Pericentrin-1 (Nup85, FROUNT) [7] was investigated. After MCP-1 treatment, Pericentrin-1 was observed re-localising to the cell periphery (Fig. 3c). In addition, after 10 min of MCP-1 treatment, CCR2 staining in permeabilised BMSCs displayed peri-nuclear staining, as previously reported [6, 7] (Fig. 3c). Investigating the kinetics of Pericentrin-1 re-localisation, we found that this occurred swiftly, between 5 and 10 min after MCP-1 treatment (Fig. 3c).

### MCP-1 signals migration through Pericentrin-1 and the βγ complex

To determine whether MCP-1 signalling required CCR2 activity, the CCR2 inhibitor (RS102895) [26] was employed. Inhibition of CCR2 activity abrogated the MCP-1-mediated re-localisation of CCR2 and Pericentrin-1 (Fig. 3d). This in turn led to a significant inhibition of MCP-1-mediated migration (Fig. 3e and Additional file 5: Figure S5a). To further probe the MCP-1-mediated signalling cascade, the role of GPCRs was investigated by using the cell-permeable peptide MPS-Phos, which sequesters the βγ complex [27]. Pre-treating mouse BMSCs with MPS-Phos abolished MCP-1-dependent redistribution of both CCR2 and Pericentrin-1 (Fig. 3f). Consistent with CCR2 inhibition, pre-treatment with more than 5 μM MPS-Phos resulted in significant inhibition of MCP-1-mediated migration (Fig. 3g and Additional file 5: Figure S5b).

### MCP-1-dependent PI3Kγ signalling

**Fig. 4:**
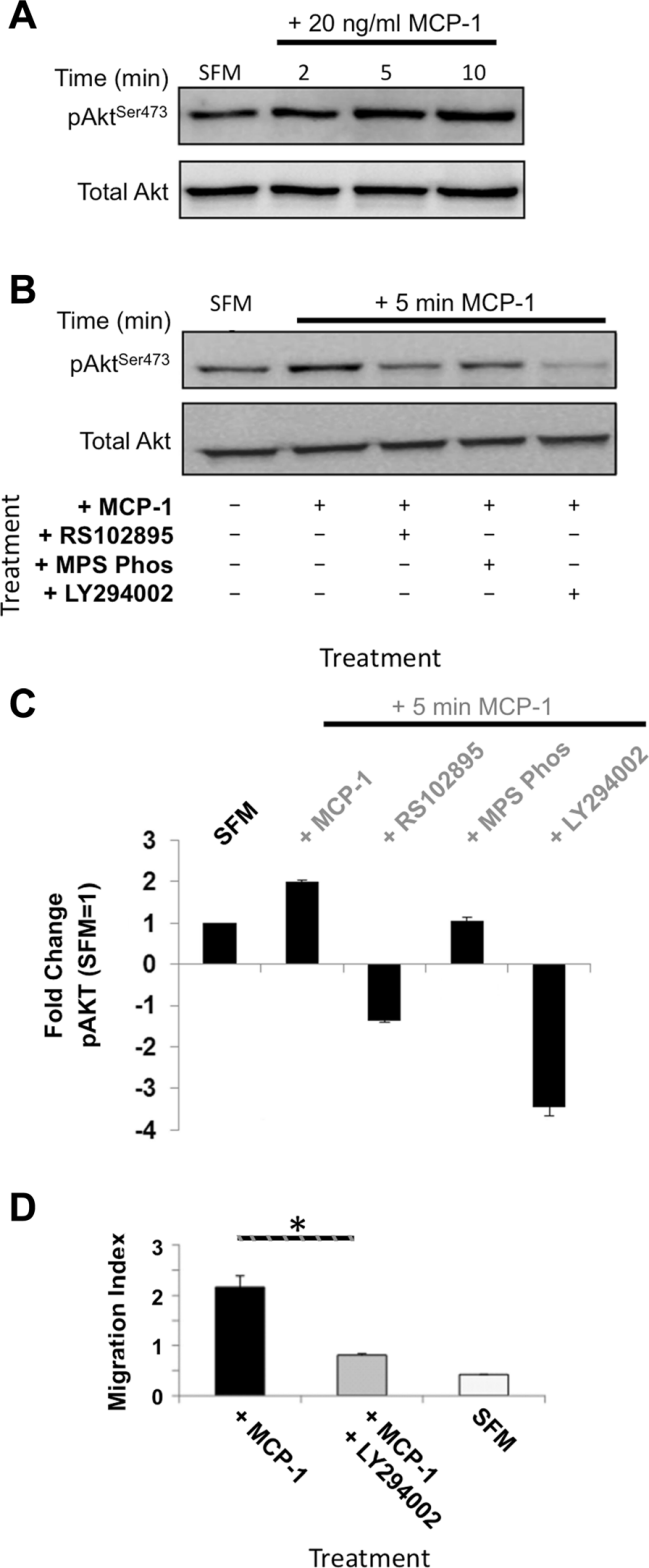
MCP-1-induced migration is dependent on GPCR βγ subunit and PI3K activity. **a** Kinetics of MCP-1-mediated AKT signalling. Immunoblotting for phosphorylated Akt^Ser473^ and total Akt following serum-starved BMSCs exposed to MCP-1 for the indicated times; 100 μg of total cell extract/lane. Representative blot of three separate independent experiments is shown. **b** Dependence of the MCP-1 signalling cascade on CCR2 and PI3Kγ activity (inhibited by 24-h serum starvation combined with 10 μM RS102895 and 10 μM LY294002, respectively). Immunoblotting for pAkt^Ser473^ and total Akt. Whole cell extracts prepared from serum-starved BMSCs pre-treated with the indicated inhibitors (10 μM RS102895, GPCR βγ subunit inhibitor 10 μM MPS-Phos, or 5 μM PI3K inhibitor LY294002) for 24 h prior to MCP-1 exposure for the indicated time; 100 μg of total cell extract/lane. Representative blot of three separate independent experiments is shown. **c** Densitometric quantification of b (Akt^Ser473^ phosphorylation). Untreated serum-starved BMSC Akt^Ser473^ levels were set to 1. Graph represents three separate independent experiments. Data are presented as mean ± standard deviation. **d** PI3Kγ activity is required for MCP-1-induced BMSC migration in three dimensions. PI3Kγ activity inhibited by 24-h pre-treatment with 5 μM LY294002. Graph represents three separate independent experiments. Data are presented as mean ± standard deviation. A *P* value of less than 0.05 (*) was deemed significant. *BMSC* bone marrow-derived stromal cell, *CCR2* chemokine (C motif) receptor 2, *GPCR* G protein-coupled receptor, *MCP-1* monocyte chemoattractant protein 1, *PI3K* PI3 kinase, *SFM* serum-free medium

To determine whether MCP-1-mediated migration required signalling though PI3K in mouse BMSCs, phosphorylation of the PI3K target AKT was examined. After MCP-1 treatment, a time-dependent increase in PI3K-dependent Akt^Ser473^ phosphorylation was observed (Fig. 4a, b). Quantification revealed that MCP-1 treatment induced a two-fold increase in phosphorylated Akt^Ser473^ (pAkt^Ser473^) (Fig. 4c, where phosphorylated Akt^Ser473^ levels following 24 h SFM media set to 1). Furthermore, combining RS102895 pre-treatment and MCP-1 resulted in a marked decrease in pAkt^Ser473^. Interestingly, pre-treatment with MPS-Phos before MCP-1 treatment did not significantly effect pAkt^Ser473^ levels, indicating that the βγ complex signals though an alternate pathway following MCP-1 treatment. To further dissect this signalling cascade, we employed the PI3K inhibitor, LY294002. Mouse BMSCs pre-treated with LY294002 had no MCP-1-induced phosphorylation of Akt^Ser473^ and indeed had pAkt^Ser473^ levels that were barely detectable (Fig. 4b, c). This reduction in pAkt^Ser473^ led to a de-sensitization of BMSCs to the effects of MCP-1, resulting in a significant inhibition of MCP-1-induced migration (Fig. 4c and Additional file 5: Figure S5c).

### The MCP-1 signalling cascade requires ERK1 phosphorylation to induce migration

**Fig. 5:**
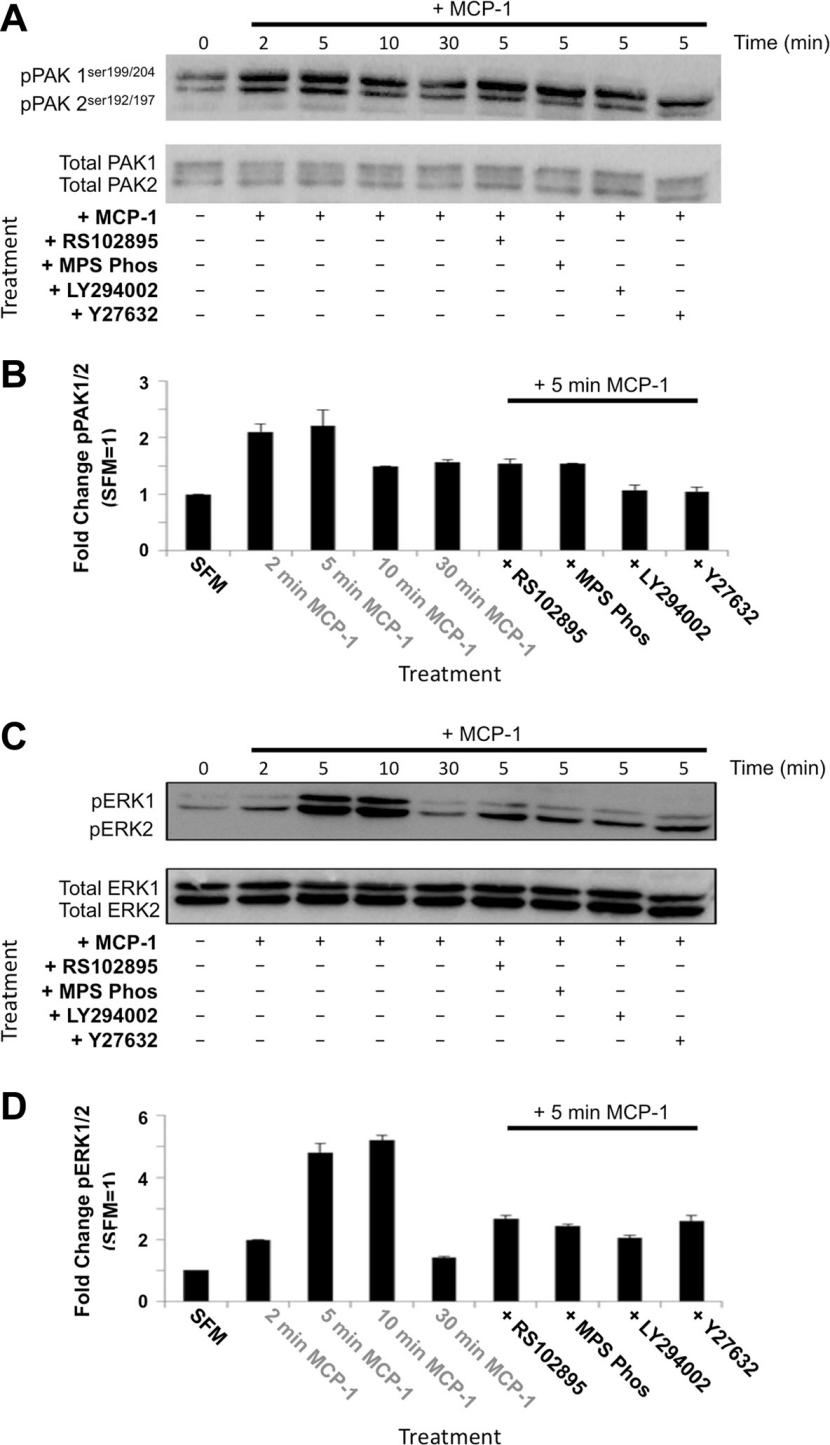
PAK and ERK activation in response to MCP-1 is dependent on GPCR βγ subunit, PI3Kγ, and ROCK activity. **a** Immunoblotting for phosphorylation of PAK1^Ser199/204^ and PAK2^Ser192/197^ and total PAK1/2. Serum starvation of BMSCs in combination with 10 μM RS102895, 10 μM MPS-Phos, 5 μM LY294002, or 10 μM Y27632 for 24 h and exposed to MCP-1 for the indicated times; 100 μg of total cell extract/lane. Representative blot of three separate independent experiments is shown. **b** Densitometric quantification of a (PAK1^Ser199/204^ and PAK2^Ser192/197^ phosphorylation). Untreated serum-starved BMSC PAK1^Ser199/204^ and PAK2^Ser192/197^ levels were set to 1. Graph represents three separate independent experiments. Data are presented as mean ± standard deviation. **c** Immunoblotting for phosphorylation of ERK1/2 and total ERK1/2 following serum-starved BMSCS was pre-treated with MCP-1 for the indicated times or with 10 μM RS102895, 10 μM MPS-Phos, 5 μM LY294002, or 10 μM Y27632 and exposed to MCP-1; 100 μg of total cell extract/lane. Representative blot of three separate independent experiments is shown. **d** Densitometric quantification of c (ERK1/2 phosphorylation). Untreated serum-starved BMSC pERK1/2 levels were set to 1. Data are presented as mean ± standard deviation. Graph represents three separate independent experiments. *BMSC* bone marrow-derived stromal cell, *ERK* extracellular signal-regulated kinase, *GPCR* G protein-coupled receptor, *MCP-1* monocyte chemoattractant protein 1, *MPS-Phos* MPS-phosducin-like protein C terminus, *PAK* P21 protein (Cdc42/Rac)-activated kinase 1, *PI3K* PI3 kinase, *ROCK* Rho-associated, coiled-coil containing protein kinase

To determine whether GPCR βγ mediated ERK activation required for MCP-1-mediated signalling in our model system, we treated mouse BMSCs with MCP-1 and evaluated the activation of PAK1 and PAK2, which are upstream of ERK activation. We found that SFM-treated cells exposed to MCP-1 displayed activation of PAK1 and PAK2 (through phosphorylation of PAK1 at Ser199/204 and PAK2 at Ser192/197) as swiftly as 2 min (Fig. 5a, b, left).

To further explore this signalling cascade, we evaluated the effect on PAK1^Ser199/204^ and PAK2^Ser192/197^ phosphorylation by combining the previously described inhibitors (RS102895, MPS-Phos, LY294002, and Y27632; Figs. 2, 3, and 4) and MCP-1 treatment (Fig. 5a, b, right side). We found that PI3K (LY294002) and ROCK (Y27632) inhibition led to a noticeable decrease in MCP-1-induced PAK2^Ser192/197^ phosphorylation (Fig. 5a). Following this trend, both CCR2 and GPCR βγ inhibition also reduced the MCP-1 induction of PAK2^Ser192/197^ phosphorylation, although quantification revealed that this was not statistically significant (Fig. 5b). Importantly, we found that 24-h SFM treatment alone, or in combination with the inhibitors, did not significantly affect BMSC viability or cytotoxicity or induce apoptosis (Additional file 6: Figure S6).

### ERK and PAK activation is required for MCP-1 stimulated migration

To further investigate the consequences of induced (activation of) PAK1 and PAK2 signalling, we explored the effects of MCP-1 treatment on the downstream molecules extracellular signal-regulated protein kinases 1 and 2 (ERK1/2). BMSCs pre-treated with SFM were exposed to MCP-1, and the kinetics of ERK1 and 2 phosphorylation (pERK1/2) were investigated (Fig. 5c). After 5 min of MCP-1 treatment, there was a marked increase in pERK1, which had almost entirely diminished by 30 min after treatment (Fig. 5c, d, left side). Interestingly, pERK2 was also strongly increased at 2 min after MCP-1 treatment, and mimicking pERK1, the pERK2 levels had returned to control levels by 30 min after treatment.

To explore the role of MCP-1 stimulation on this signalling cascade, we evaluated the effects of the previously described inhibitors (RS102895, MPS-Phos, LY294002, and Y27632; Figs. 2, 3, and 4) in combination with MCP-1 treatment. Accompanying the results of inhibition of pPAK1/2, we observed a reduction in the levels of pERK1/2 (Fig. 5c, d, right side). Supporting previous reports in human cells [28, 29], Y27632 pre-treatment combined with MCP-1 led to a decrease in pERK1/2. However, there are conflicting reports indicating that Y27632 did not affect phosphorylation of ERK1 and 2 in human leukocytes [30].

## Discussion

The ability of stem cells to migrate provides the basis for the therapeutic treatment of a broad spectrum of conditions via systemic infusion of *ex vivo* preparations of stem cells. To maximize their therapeutic efficacy, an understanding of the various molecular mechanisms and signalling cascades mediating stem cell migration is essential [8, 31]. Furthermore, characterising the differences in migration mechanisms between human and the model organism mouse is essential to allow results from *in vivo* models to be accurately and meaningfully translated into human trials.

**Fig. 6:**
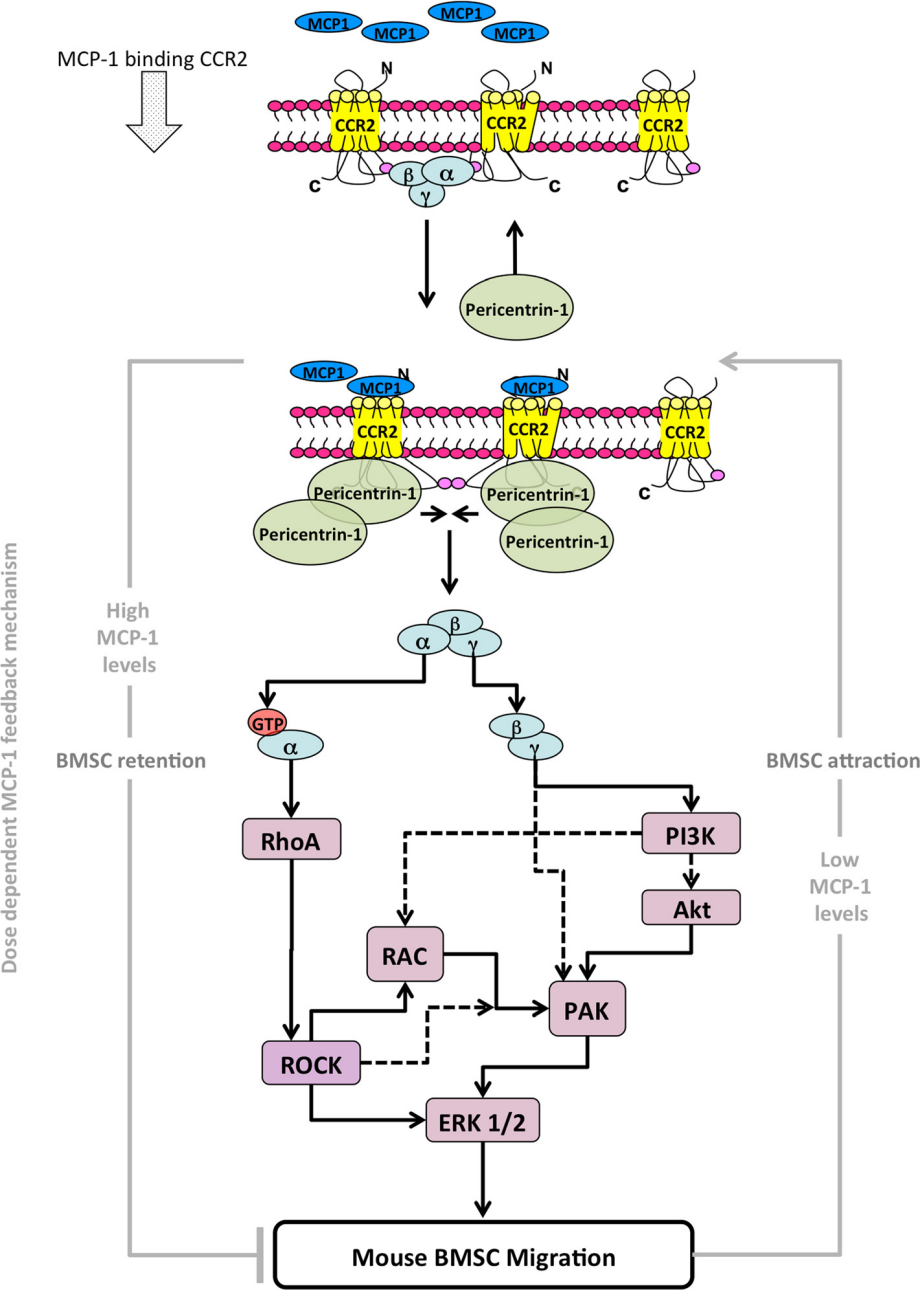
Proposed model of MCP-1-dependent signalling cascade in mouse BMSCs. Solid lines indicate direct interactions. Dotted lines indicate indirect actions. *BMSC* bone marrow-derived stromal cell, *ERK* extracellular signal-regulated kinase, *MCP-1* monocyte chemoattractant protein 1, *PAK* P21 protein (Cdc42/Rac)-activated kinase 1, *PI3K* PI3 kinase, *Rac* Ras-related C3 botulinum toxin substrate 1, *RhoA* rhodopsin A, *ROCK* Rho-associated, coiled-coil containing protein kinase

Here, we have highlighted key differences in the way mouse BMSCs respond and signal in response to the key chemokine MCP-1 (Fig. 6). In addition, we have revealed previously unknown insights into the timing of these signalling cascades and characterised the key players in the cascades that facilitate mouse BMSC migration in response to chemokines (Figs. 3, 4, and 5). Interestingly, we found that chemotactic migration of mouse BMSCs requires ROCK activity (Fig. 2), in contrast to reports in other organisms (human, chicken, rat, and mouse) and cell types (glioma, mouse embryonic fibroblast, chick embryonic heart fibroblast, MSCs, and breast cancer) [12, 14, 20, 23–25]. Intriguingly, our findings were supported by other studies but only in human cells [10, 28, 30, 32, 33]. Significantly, in response to MCP-1, ROCK-inhibited BMSCs were unable to migrate in a 3D assay and their ability to migrate in a 2D assay was also severely reduced (Fig. 2b, c). Interestingly, we found that BMSCs displayed an MCP-1 dose-dependent inhibition of migration (Additional file 2: Figure S2b). This suggests the presence of a positive feedback loop mechanism, where lower concentrations of MCP-1 attract BMSCs and the higher concentrations encountered at the site of MCP-1 secretion result in a settling and retention of BMSCs. Indeed, the MCP-1 gradient used in our study would include the reported plasma levels of MCP-1 in healthy individuals (0.1–0.7 ng/ml) [34–36]. Supporting this, we found that the majority of migrating cells were observed at the lower end of the MCP-1 gradient (data not shown). It is possible that acute exposure to MCP-1 alters the differentiation potential of BMSCs; however, owing to the extremely short time frames investigated, additional experimentation would be required to confirm this.

To investigate the signalling cascades involved in mouse BMSC migration, we began with MCP-1 ligand binding and receptor activation and tracked this signalling, which ultimately results in cytoskeletal reorganisation and migration. We found that this sequence of events, including the clustering of Pericentrin-1, is entirely dependent on the release and activation of the βγ subunits from the Gαβγ receptor complex. This is supported by our findings that βγ subunit activation is required for BMSC migration in response to MCP-1. Importantly, we demonstrate that the chemotactic migration cascade is initiated with CCR2 re-localisation, supporting previous findings [37]. However, this activation does not affect expression, particularly in such short time frames (Fig. 3).

Importantly, we defined the previously unknown kinetics of this signalling cascade, and using specific inhibitors we were able to characterise the key players in mouse BMSC chemotactic migration (Figs. 3, 4, and 5). We demonstrate that ROCK inhibition in BMSCs significantly retards migration which is likely through the observed disruption of ERK1/2 phosphorylation; however, this remains to be verified directly by ERK1/2 inhibition. This is a novel finding which is in contrast to a previous report of ROCK inhibition which, while inhibiting chemotactic migration, did not affect ERK1/2 phosphorylation [30]. It is interesting to note that the underlying signalling was different, which may represent a fundamental difference in the cell types (mouse BMSCs versus human leukocytes), chemokine (fMLP versus MCP-1) or receptor (FRP1 versus CCR2). Further work will need to focus on different chemokines that induce BMSC migration, such as transforming growth factor-beta, and determine whether they share common signalling pathways. Our work has highlighted the importance of determining whether these pathways are conserved in human BMSCs, which may impact on any therapeutic applications.

We found that, in BMSCs, PI3Kγ plays a pivotal role in chemotactic-induced signalling and migration, supporting previous work in monocytes and macrophages [9, 38]. Importantly, our data show that the transient phosphorylation of ERK succeeds the transient phosphorylation of PAK (Fig. 5). This signalling is important for defining the leading edge of the cell in response to a chemotactic signal, as PI3Ks contribute to cell polarity and migration by regulation of the downstream RhoGTPase, Rac. Activated Rac (Rac^GTP^) then interacts with several downstream effectors, PAK being one of them, to coordinate cytoskeletal reorganization, lamellipodia formation, and resultant migration [39, 40]. Although active Rac (through ERK and PAK) and associated signalling is involved in orchestrating cytoskeletal dynamics at the leading edge of the cell, Rho/ROCK has largely been implicated in retraction at the rear of migrating cells [10, 30, 41, 42]. However, recent reports have confirmed that Rho/ROCK signalling is also active in lamellipodia [22, 43, 44].

## Conclusions

Our findings advance our understanding of bone marrow stromal (mesenchymal stem) cell biology and the mechanisms orchestrating chemotactic migration. Using a systematic approach, we mapped additional components to the chemokine signalling pathway and provide additional novel data concerning the timing of these signals (Fig. 6). Furthermore, for the first time, our work provides a detailed mechanistic analysis of motility signalling in BMSCs. This is significant, as increasing our knowledge of the chemokine-dependent signalling mechanisms essential for migration is undeniably required to fully exploit any therapeutic applications. In addition, we define and highlight key areas where the model organism *Mus musculus* differs from humans, which may affect the translation of therapeutic applications. Finally, we have described potential additional therapeutic targets in BMSCs that could be exploited to improve delivery, homing, and retention of BMSCs to sites of injury or disease.

## Additional files

Additional file 1: Figure S1. Stages of BMSCs observed in culture. Permeabilised serum-starved BMSCs immunofluorescently stained with F-actin (red) and α-tubulin (green) and DNA counter-stained with DAPI (blue). Scale bar = 10 μm. Representative BMSC stages observed are immotile, polarised, and actively motile. F-Actin filaments accumulate at the leading edge in polarised BMSCs. Actively motile BMSCs display an accumulation of F-Actin and α-tubulin at the cell edge orientated in the direction of movement. Isotype controls, mouse and rabbit (Thermo Fisher Scientific), demonstrate specificity of staining. *BMSC* bone marrow-derived stromal cell, *DAPI* 4′,6-diamidino-2-phenylindole. (PNG 558 kb) Additional file 2: Figure S2. MCP-1 induced significant BMSC migration. **a** Immunofluorescent images of representative fields of serum-starved (24 h) BMSCs migrating in response to a gradient of MCP-1 in a 2D assay. Permeabilised serum-starved BMSCs immunofluorescently stained with F-actin (red) and α-tubulin (green) and DNA stained with DAPI (blue). Scale bar = 30 μm. **b** BMSC migration in response to the indicated MCP-1 concentrations in a 3D migration assay. Graph represents three separate independent experiments. Data are presented as mean ± SD. A *P* value of less than 0.05 (*) was deemed significant. **c** Representative 3D migration assay results from serum-starved BMSCs exposed to the indicated conditions. *2D* two-dimensional, *3D* three-dimensional, *BMSC* bone marrow-derived stromal cell, *DAPI* 4′,6-diamidino-2-phenylindole, *MCP-1* monocyte chemoattractant protein 1, *SD* standard deviation. (PNG 571 kb) Additional file 3: Figure S3. ROCK inhibition affects morphology and MCP-1-induced migration in mouse BMSCs. Permeabilised serum-starved BMSCs immunofluorescently stained with F-actin (red) and α-tubulin (green) and DNA counter-stained with DAPI (blue). **a** Serum-starved BMSCs polarise in response to MCP-1; 10 μM Y27632 pre-treatment (24 h) results in a stellate morphology, with or without MCP-1 treatment. Scale bar = 10 μm. **b** Immunofluorescent images of representative fields of serum-starved (24 h) BMSCs pre-treated with 10 μM Y27632 and exposed to a gradient of MCP-1 in a 2D assay. Permeabilised serum-starved BMSC stained with F-actin (red) and α-tubulin (green) and DNA stained with DAPI (blue). Scale bar = 30 μm. *2D* two-dimensional, *BMSC* bone marrow-derived stromal cell, *DAPI* 4′,6-diamidino-2-phenylindole, *MCP-1* monocyte chemoattractant protein 1, *ROCK* Rho-associated, coiled-coil containing protein kinase. (PNG 1480 kb) Additional file 4: Figure S4. Human BMSC chemotactic migration is unaffected by ROCK inhibition but does display the characteristic morphology. **a** Serum-starved human BMSCs polarise in response to MCP-1; 10 μM Y27632 treatment results in stellate morphology in BMSCs, independent of MCP-1 treatment. Scale bar = 10 μm. **b** ROCK inhibition does not affect human BMSC migration in response to MCP-1. Immunofluorescent images of representative fields of serum-starved (24 h) BMSCs pre-treated with 10 μM Y27632 and exposed to a gradient of MCP-1 in a 2D assay. Permeabilised serum-starved BMSCs stained with F-actin (red) and α-tubulin (green) and DNA stained with DAPI (blue). Scale bar = 30 μm. **c** Quantification of **b**. Graph represents two separate independent experiments (>100 cells evaluated for each separate independent experiment). Data are presented as mean ± standard error of the mean. A *P* value of less than 0.05 (*) was deemed significant, and a *P* <0.001 (***) highly significant. *2D* two-dimensional, *BMSC* bone marrow-derived stromal cell, *DAPI* 4′,6-diamidino-2-phenylindole, *MCP-1* monocyte chemoattractant protein 1, *ROCK* Rho-associated, coiled-coil containing protein kinase. (PNG 1175 kb) Additional file 5: Figure S5. CCR2, GPCR βγ subunit and PI3K activity is required for MCP-1-induced BMSC migration. **a**-**c** 3D migration of serum-starved BMSCs pre-treated with 10 μM RS102895, 10 μM MPS-Phos, 5 μM LY294002, or 10 μM Y27632 for 24 h prior to exposure to MCP-1. Representative migration traces shown of three separate independent experiments; 10 % fetal bovine serum included as a positive control. *3D* three-dimensional, *BMSC* bone marrow-derived stromal cell, *CCR* chemokine (C motif) receptor, *GPCR* G protein-coupled receptor, *MCP-1* monocyte chemoattractant protein 1, *PI3K* PI3 kinase. (PNG 273 kb) Additional file 6: Figure S6. Inhibitors do not affect BMSC viability or cytotoxicity or induce apoptosis; 24-h serum starvation (SFM) of BMSCs alone or in conjunction with 10 μM Y27632, 5 μM LY294002, 10 μM MPS-Phos, or 0.5 μM Staurosporine. A very low dose of Staurosporine was included as a positive control for apoptosis. Untreated cells (cultured in normal media) were included as an untreated control. SFM set = 1. Graph represents three independent experiments. (Each individual experiment was performed in triplicate.) Data are presented as mean ± standard error of the mean. A *P* value of less than 0.05 (*) was deemed significant, and a *P* value of less than 0.01 (**) very significant. *BMSC* bone marrow-derived stromal cell, *SFM* serum-free medium. (PNG 360 kb)

## Abbreviations

2D: Two-dimensional
3D: Three-dimensional
BMSC: Bone marrow-derived stromal cell
CCR: Chemokine (C motif) receptor
ERK: Extracellular signal-regulated kinase
FCS: Fetal calf serum
GPCR: G protein-coupled receptors
MCP-1: Monocyte chemoattractant protein 1
MEM-α: Modified Eagle’s medium-α
MI: Migration index
MPS-Phos: MPS-phosducin-like protein C terminus
MSC: Mesenchymal stem cell
PAK: P21 Protein (Cdc42/Rac)-activated kinase 1
PI3K: PI3 kinase
Rac: Ras-related C3 botulinum toxin substrate 1
Rho: Rhodopsin
ROCK: Rho-associated, coiled-coil containing protein kinase
SFM: Serum-free medium
