# Supplementary figures and images for: ROCK activity and the Gβγ complex mediate chemotactic migration of mouse bone marrow-derived stromal cells
Source: Stem Cell Res Ther. 2015 Jul 24;6:136. doi: 10.1186/s13287-015-0125-y (PMC4603944; doi:10.1186/s13287-015-0125-y)

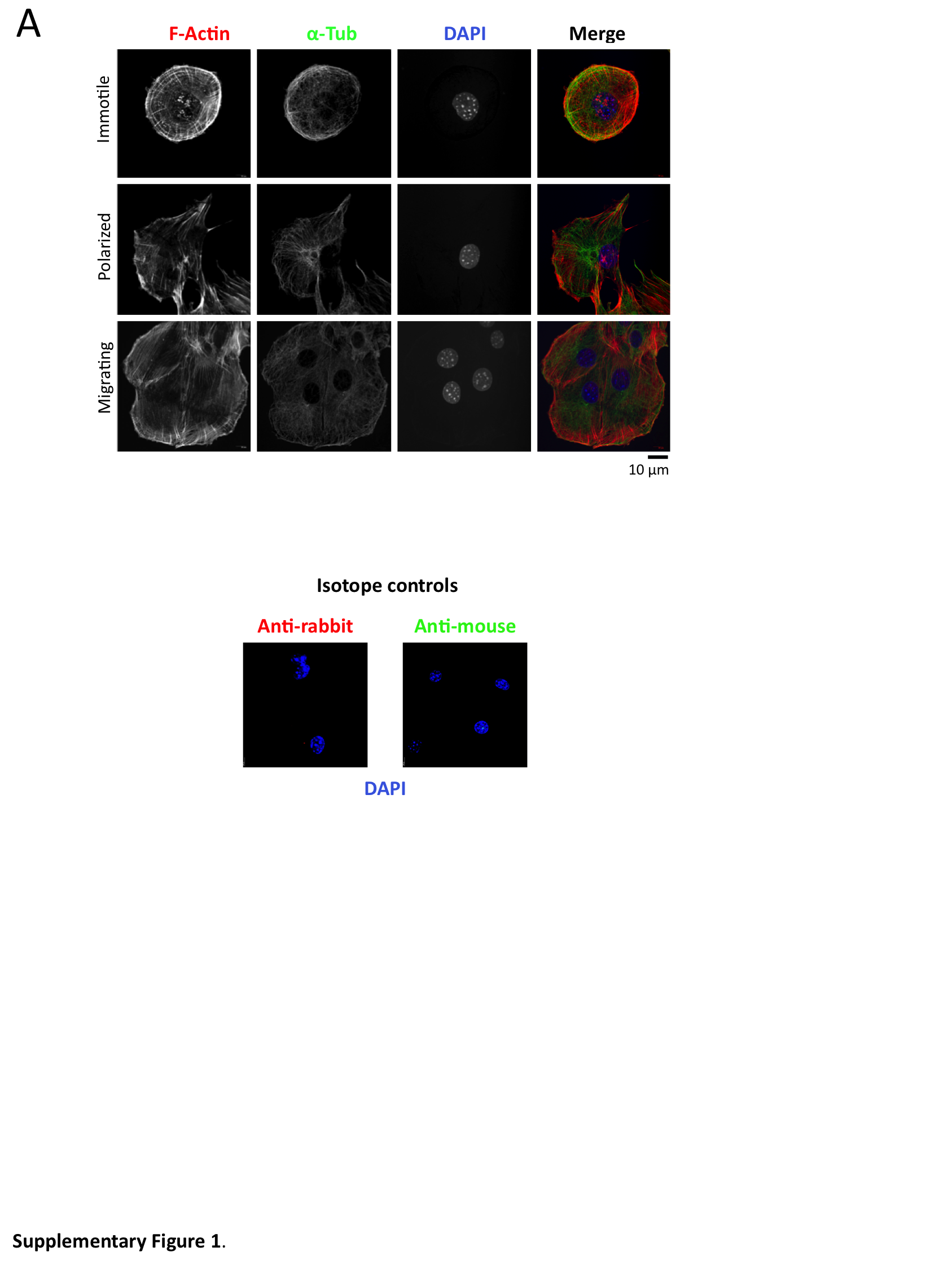

Supplement: Additional file 1: Figure S1. — Stages of BMSCs observed in culture. Permeabilised serum-starved BMSCs immunofluorescently stained with F-actin (red) and α-tubulin (green) and DNA counter-stained with DAPI (blue). Scale bar = 10 μm. Representative BMSC stages observed are immotile, polarised, and actively motile. F-Actin filaments accumulate at the leading edge in polarised BMSCs. Actively motile BMSCs display an accumulation of F-Actin and α-tubulin at the cell edge orientated in the direction of movement. Isotype controls, mouse and rabbit (Thermo Fisher Scientific), demonstrate specificity of staining. BMSC bone marrow-derived stromal cell, DAPI 4′,6-diamidino-2-phenylindole. (PNG 558 kb) [file 13287_2015_125_MOESM1_ESM.png]

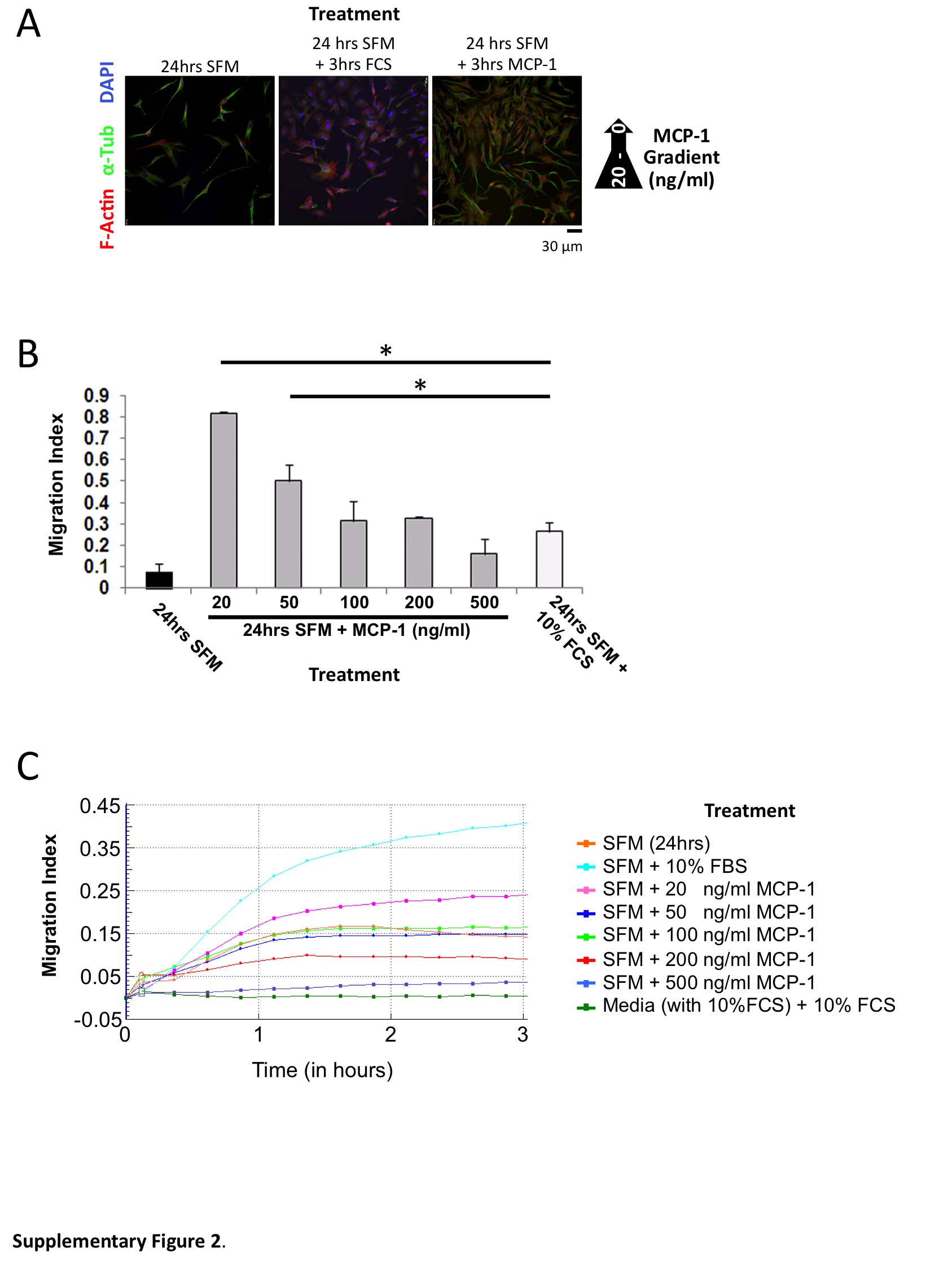

Supplement: Additional file 2: Figure S2. — MCP-1 induced significant BMSC migration. a Immunofluorescent images of representative fields of serum-starved (24 h) BMSCs migrating in response to a gradient of MCP-1 in a 2D assay. Permeabilised serum-starved BMSCs immunofluorescently stained with F-actin (red) and α-tubulin (green) and DNA stained with DAPI (blue). Scale bar = 30 μm. b BMSC migration in response to the indicated MCP-1 concentrations in a 3D migration assay. Graph represents three separate independent experiments. Data are presented as mean ± SD. A P value of less than 0.05 (*) was deemed significant. c Representative 3D migration assay results from serum-starved BMSCs exposed to the indicated conditions. 2D two-dimensional, 3D three-dimensional, BMSC bone marrow-derived stromal cell, DAPI 4′,6-diamidino-2-phenylindole, MCP-1 monocyte chemoattractant protein 1, SD standard deviation. (PNG 571 kb) [file 13287_2015_125_MOESM2_ESM.png]

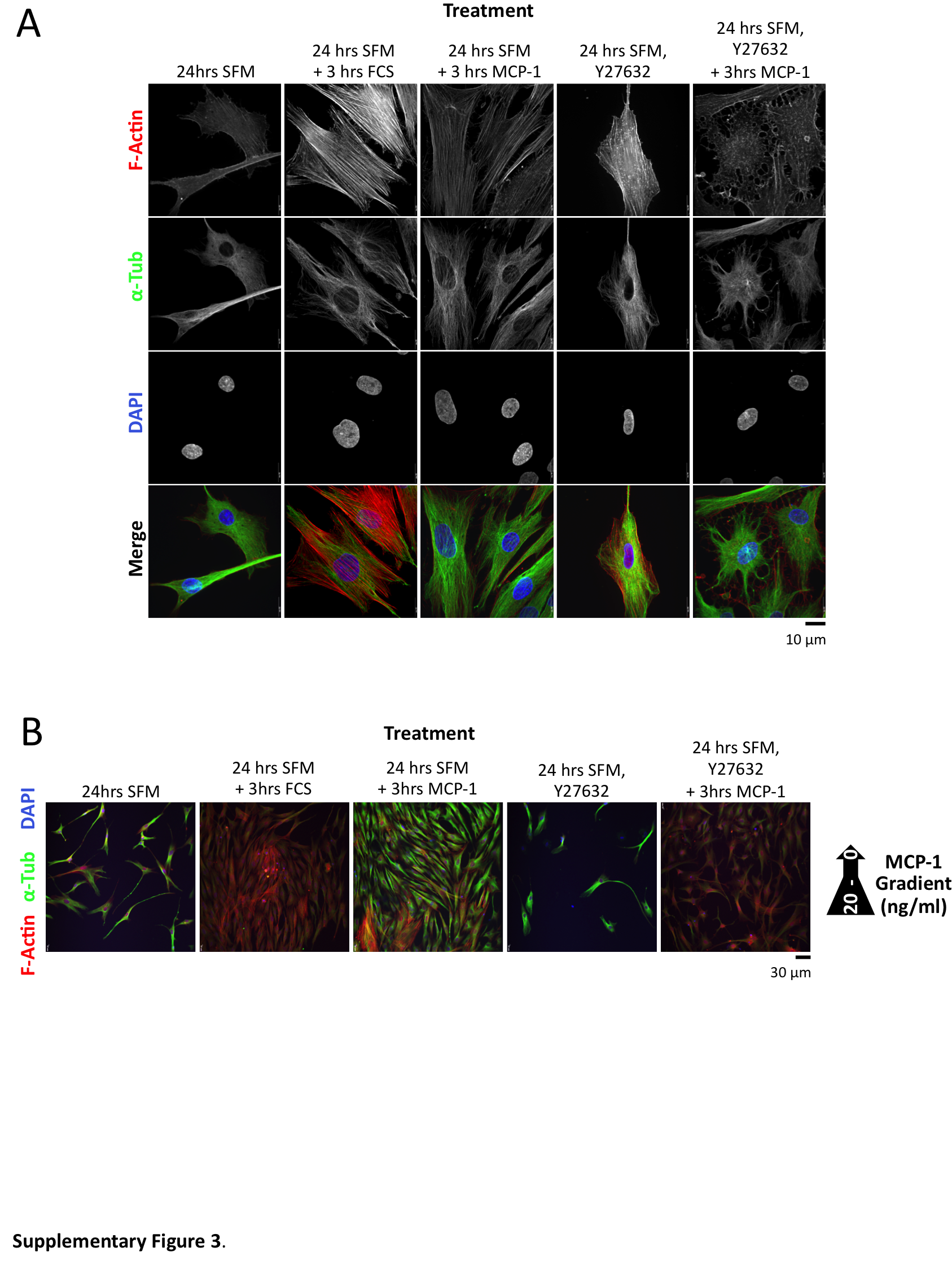

Supplement: Additional file 3: Figure S3. — ROCK inhibition affects morphology and MCP-1-induced migration in mouse BMSCs. Permeabilised serum-starved BMSCs immunofluorescently stained with F-actin (red) and α-tubulin (green) and DNA counter-stained with DAPI (blue). a Serum-starved BMSCs polarise in response to MCP-1; 10 μM Y27632 pre-treatment (24 h) results in a stellate morphology, with or without MCP-1 treatment. Scale bar = 10 μm. b Immunofluorescent images of representative fields of serum-starved (24 h) BMSCs pre-treated with 10 μM Y27632 and exposed to a gradient of MCP-1 in a 2D assay. Permeabilised serum-starved BMSC stained with F-actin (red) and α-tubulin (green) and DNA stained with DAPI (blue). Scale bar = 30 μm. 2D two-dimensional, BMSC bone marrow-derived stromal cell, DAPI 4′,6-diamidino-2-phenylindole, MCP-1 monocyte chemoattractant protein 1, ROCK Rho-associated, coiled-coil containing protein kinase. (PNG 1480 kb) [file 13287_2015_125_MOESM3_ESM.png]

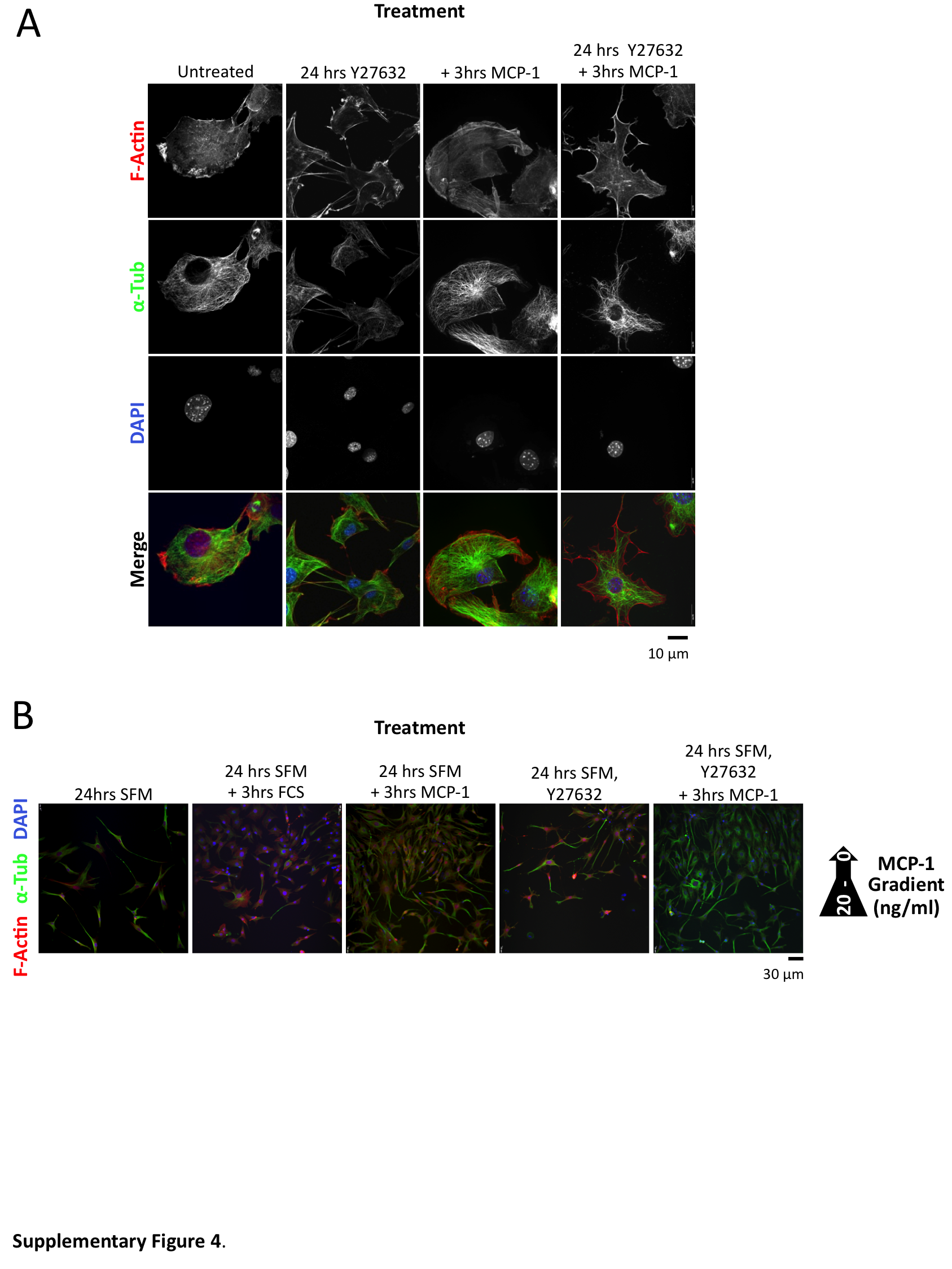

Supplement: Additional file 4: Figure S4. — Human BMSC chemotactic migration is unaffected by ROCK inhibition but does display the characteristic morphology. a Serum-starved human BMSCs polarise in response to MCP-1; 10 μM Y27632 treatment results in stellate morphology in BMSCs, independent of MCP-1 treatment. Scale bar = 10 μm. b ROCK inhibition does not affect human BMSC migration in response to MCP-1. Immunofluorescent images of representative fields of serum-starved (24 h) BMSCs pre-treated with 10 μM Y27632 and exposed to a gradient of MCP-1 in a 2D assay. Permeabilised serum-starved BMSCs stained with F-actin (red) and α-tubulin (green) and DNA stained with DAPI (blue). Scale bar = 30 μm. c Quantification of b. Graph represents two separate independent experiments (>100 cells evaluated for each separate independent experiment). Data are presented as mean ± standard error of the mean. A P value of less than 0.05 (*) was deemed significant, and a P <0.001 (***) highly significant. 2D two-dimensional, BMSC bone marrow-derived stromal cell, DAPI 4′,6-diamidino-2-phenylindole, MCP-1 monocyte chemoattractant protein 1, ROCK Rho-associated, coiled-coil containing protein kinase. (PNG 1175 kb) [file 13287_2015_125_MOESM4_ESM.png]

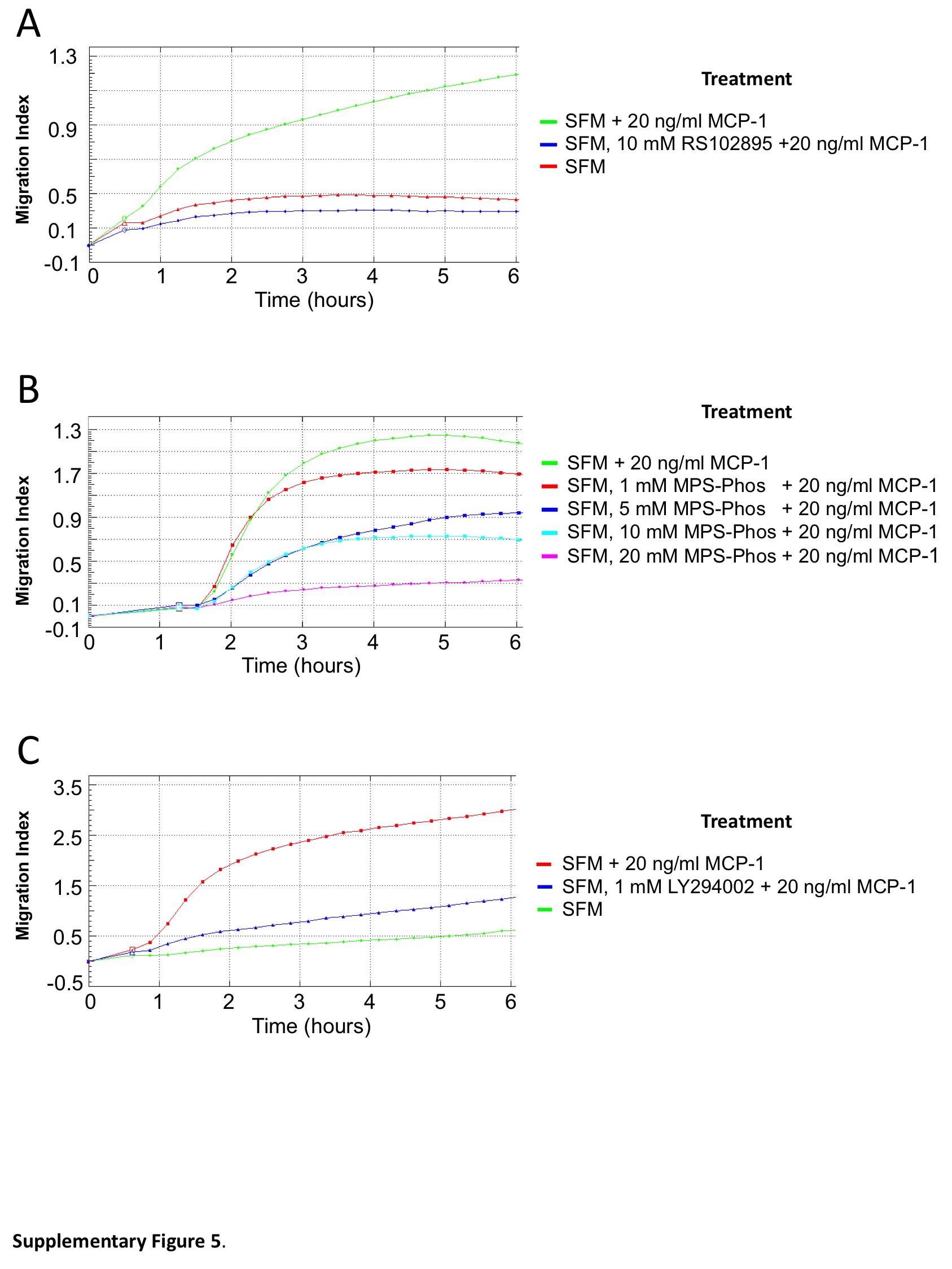

Supplement: Additional file 5: Figure S5. — CCR2, GPCR βγ subunit and PI3K activity is required for MCP-1-induced BMSC migration. a-c 3D migration of serum-starved BMSCs pre-treated with 10 μM RS102895, 10 μM MPS-Phos, 5 μM LY294002, or 10 μM Y27632 for 24 h prior to exposure to MCP-1. Representative migration traces shown of three separate independent experiments; 10 % fetal bovine serum included as a positive control. 3D three-dimensional, BMSC bone marrow-derived stromal cell, CCR chemokine (C motif) receptor, GPCR G protein-coupled receptor, MCP-1 monocyte chemoattractant protein 1, PI3K PI3 kinase. (PNG 273 kb) [file 13287_2015_125_MOESM5_ESM.png]

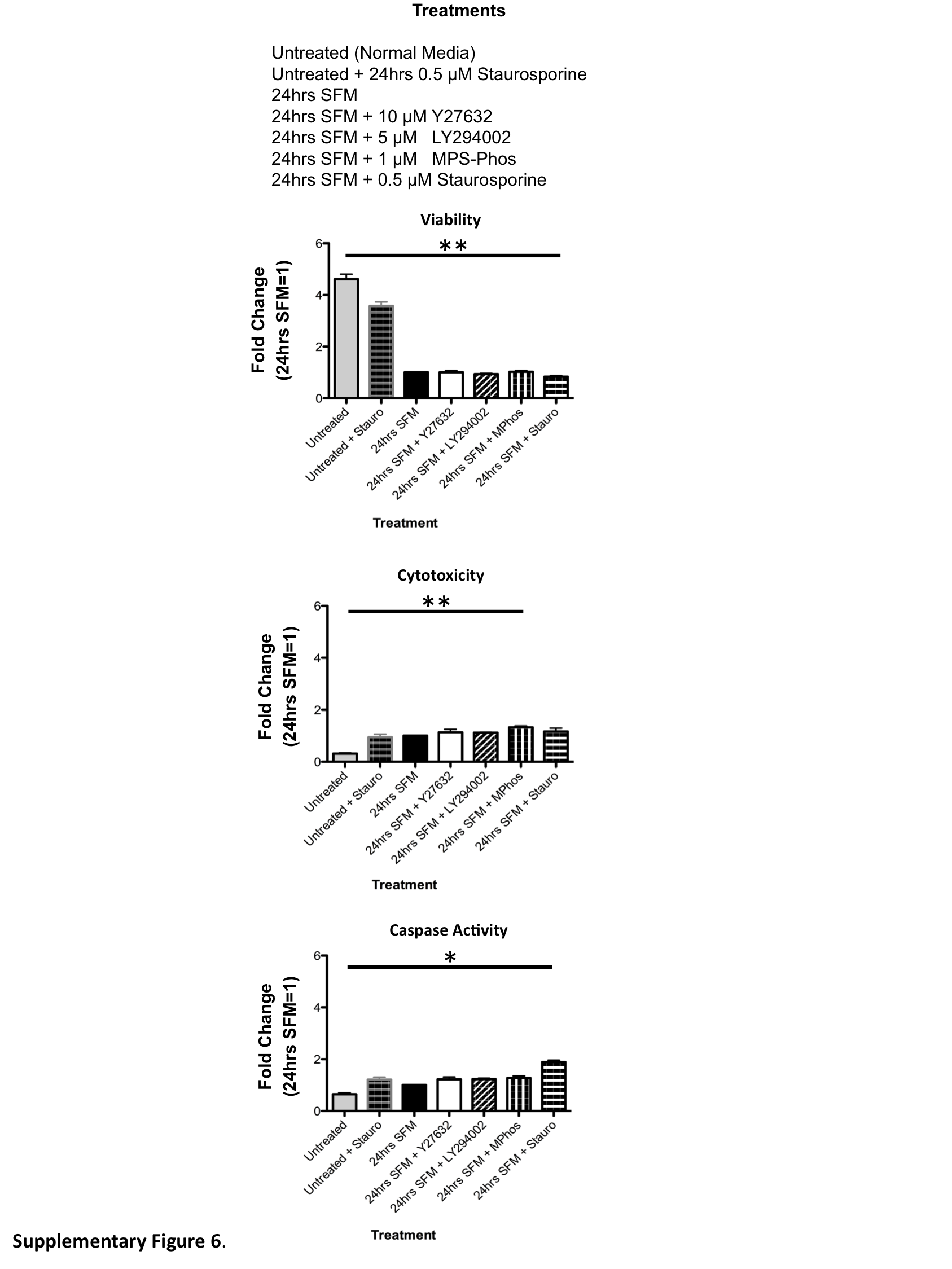

Supplement: Additional file 6: Figure S6. — Inhibitors do not affect BMSC viability or cytotoxicity or induce apoptosis; 24-h serum starvation (SFM) of BMSCs alone or in conjunction with 10 μM Y27632, 5 μM LY294002, 10 μM MPS-Phos, or 0.5 μM Staurosporine. A very low dose of Staurosporine was included as a positive control for apoptosis. Untreated cells (cultured in normal media) were included as an untreated control. SFM set = 1. Graph represents three independent experiments. (Each individual experiment was performed in triplicate.) Data are presented as mean ± standard error of the mean. A P value of less than 0.05 (*) was deemed significant, and a P value of less than 0.01 (**) very significant. BMSC bone marrow-derived stromal cell, SFM serum-free medium. (PNG 360 kb) [file 13287_2015_125_MOESM6_ESM.png]
